# Measuring the Urban Particulate Matter Island Effect with Rapid Urban Expansion

**DOI:** 10.3390/ijerph17155535

**Published:** 2020-07-31

**Authors:** Yu Cao, Xiaoqian Fang, Jiayi Wang, Guoyu Li, Yu Cao, Yan Li

**Affiliations:** Department of Land Management, School of Public Affairs, Zhejiang University, Hangzhou 310058, China; caoyu@zju.edu.cn (Y.C.); fangxiaoqian@zju.edu.cn (X.F.); 21822128@zju.edu.cn (J.W.); liguoyu@zju.edu.cn (G.L.); caoyu98@zju.edu.cn (Y.C.)

**Keywords:** urban particulate matter island (UPI) effect, air pollution, urban expansion, Hangzhou Bay area

## Abstract

Rapid urbanization has posed numerous negative impacts on the environment, including fine particulate matter (PM_2.5_) pollution. However, quantitative investigations of the PM_2.5_ concentration trends over an urban-rural gradient at the local level are still lacking. The urban particulate matter island (UPI) effect, representing the phenomenon that high particle concentrations in urban areas are gradually attenuated to surrounding areas, was adopted and modified in this paper to study the Hangzhou Bay area from 2000 to 2015. We found the following: (1) every urban area in the Hangzhou Bay area experienced rapid expansion, especially during 2000–2005; (2) more than half of the urban areas suffered UPI problems, and these urban areas had relatively high and stable UPI intensity (UPII) values, although the UPI footprint (UPIFP) values decreased with urban expansion; and (3) urban areas could be divided into three categories: plain areas, hilly areas and the junction of plains and hills, and the probability of the UPI effect varied significantly for different categories. This paper can compensate for the lack of research on the UPI effect at the local level and provide scientific evidence for air pollution control during urban agglomeration planning.

## 1. Introduction

Urbanization, a major anthropogenic modification of Earth, occurs at an unprecedented speed in recorded human history worldwide [1,2]. According to the Department of Economic and Social Affairs of the United Nations Secretariat, more than half of the global population currently lives in urban areas (55%, up from 30% in 1950), and by the middle of the century, the proportion is projected to reach 68%. Generally, no other anthropogenic activity alters the environment more radically and persistently than urbanization, and air pollution [3], the urban heat island (UHI) effect [4], global climate change [5], etc. have been proven to be related to rapid urbanization. Among these alarming problems, fine particulate matter with an aerodynamic diameter smaller than 2.5 μm (PM_2.5_) has elicited considerable public attention because of its harmful effects on public health. There is convincing evidence that PM_2.5_ can cause respiratory and cardiovascular problems, lung disease and other health problems [6,7]. Therefore, obtaining a better understanding of PM_2.5_ pollution control is of great importance. Many studies have investigated meteorological [8,9] and socioeconomic factors [10,11] that may influence the spatiotemporal dynamics of the PM_2.5_ concentration.

At the same time, urban land has extensively expanded outward across global cities to meet the needs of the soaring number of city dwellers [1]. A worldwide meta-analysis performed by Seto et al. [12] indicated that the urban land area had increased by 58,000 km^2^ from 1970 to 2000, with the highest rate of urban expansion observed in India, China, and Africa. Spatiotemporal analysis of the urban expansion pattern and dynamics is important not only in urban geography but also in urban planning, environmental, and sustainability studies [13]. Many methods for measuring urban expansion have been proposed. Among these methods, quantitative metrics generated through the size and distribution of new urban land based on land cover maps have been widely applied to assess the physical growth of cities [14,15].

It has been proven that rapid urban expansion exerts many negative impacts on the environment that can transcend far beyond its physical boundary [16]. Among these impacts, the Urban Heat Island (UHI) effect, which is the phenomenon whereby urban areas tend to have a higher temperature than surrounding areas, has been relatively widely researched [2]. In addition to heat energy, cities are also considered a major container of air pollutants. A previous study found that PM_2.5_ pollution presented similar characteristics to the temperature; thus, a new concept—the urban particulate matter island (UPI) effect—was introduced. Its definition is the phenomenon whereby a higher particle concentration is observed in urban areas that is gradually attenuated to surrounding areas. In addition, two metrics—UPII (UPI intensity) and UPIFP (UPI footprint)—were designed to quantitatively delineate the UPI phenomenon of 338 prefectures throughout China [17].

PM_2.5_ data can be divided into two categories—ground monitoring site data and remote sensing data—according to the data source. For the data obtained from ground monitoring sites in China, the PM_2.5_ concentration was incorporated in the monitoring system by the National Atmospheric Environment Monitoring System in 2012, and national monitoring of the PM_2.5_ concentration was absent until 2013. Therefore, it is not feasible to use ground observation data for studies on the PM_2.5_ pollution in China before 2013. In addition, due to the uneven coverage of the ground monitoring sites, limited data are available for studying the spatial and temporal distribution features of PM_2.5_. Luckily, satellite-based observations can complement the existing network of ground monitoring stations. PM_2.5_ concentrations, however, cannot be measured directly by satellite imagery. Numerous studies have demonstrated that the aerosol optical depth (AOD) obtained by using satellite instruments, such as Moderate-resolution Imaging Spectroradiometer (MODIS) and Multi-angle Imaging Spectroradiometer (MISR), exhibited a notable correlation with surface PM_2.5_ concentrations through statistical and mathematical models [18,19,20,21]. Owing to the improvements in remote sensing technology, the PM_2.5_ concentration data estimated by satellite AOD products have been widely used in research [22,23,24].

However, the rationale of the UPI effect and the interaction between cities have been ignored in previous research. To improve the model of the UPI effect, three areas (urban, surrounding, and background areas) and three PM_2.5_ processes (emission, dispersion, and deposition) are investigated in this paper. Therefore, all analyses of the UPI effect can be pinpointed to specific areas and processes. The surrounding and background areas constantly changed with the urban area due to urban expansion. Moreover, studies on the UPI effect at the local level are still lacking. To bridge this gap, the Hangzhou Bay area, an urban agglomeration that has experienced rapid urbanization and has suffered severe PM_2.5_ pollution over the past two decades, was selected as the study area. Considering that the county or district of every prefecture is the most basic economic and social unit in China, the UPI effect of every county or district in the Hangzhou Bay area was studied in this paper. The Hangzhou Bay area is an area with many cultural heritages, such as the West Lake, Liangzhu cultural sites, and numerous museums. PM_2.5_ pollution can have a certain impact on cultural heritage. Therefore, this paper analyzed the pollution island effect of the Hangzhou Bay area on a relatively macro scale. In this study, a total of 27 urban areas were extracted from the Hangzhou Bay area in 2000, 2005, 2010, and 2015. Then, the occurrence of the UPI effect was determined—the urban areas whose PM_2.5_ concentration gradually attenuated to the surrounding buffer zones were regarded as areas experiencing the UPI effect. In addition, the spatiotemporal dynamics of UPII and UPIFP were analyzed. Finally, we evaluated the results in terms of the emission, dispersion, and deposition processes. This study aims to answer the following three questions: (1) does the UPI effect exist in the Hangzhou Bay area? (2) If so, what is its magnitude and extent? (3) Is there any regularity in the UPI effect?

By dividing the study area into urban, surrounding, and background areas, this paper regarded the study area as a whole. By dividing the dynamic processes of pollutants into emission, dispersion, and deposition, this paper analyzed the UPI effect at the process level. By choosing an urban agglomeration as the study area, the UPI effect was studied at the local level. Although the Hangzhou Bay area is just a small part of eastern China, it is a typical urban agglomeration suffering severe PM_2.5_ pollution, with both plains and hills. Hence, this paper is not just a case study in the Hangzhou Bay area, because the methods can be widely applied to air pollution control during urban agglomeration planning. Compared with existing models, the modified model in this paper can help to analyze the spatial heterogeneity of the UPI effect from the mechanism perspective.

## 2. Study Area and Materials

### 2.1. Study Area

**Figure 1:**
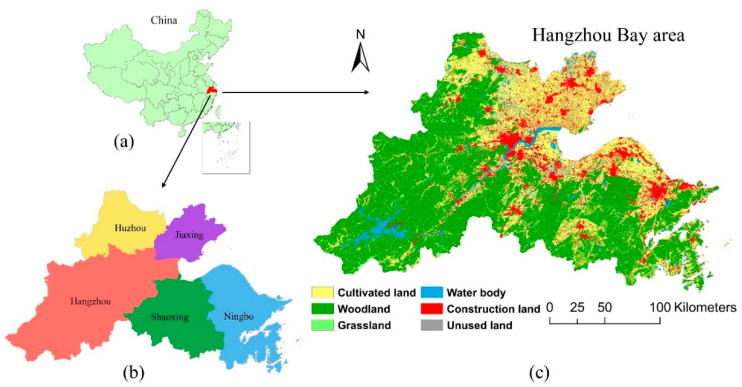
(**a**) Geographical location of the Hangzhou Bay area; (**b**) five prefecture-level cities in the Hangzhou Bay area; (**c**) land use of the Hangzhou Bay area in 2015.

Hangzhou Bay is in the coastal area of southeastern China (Figure 1a), which is both the bay and the entrance to the Qiantang River. In this paper, the Hangzhou Bay area refers to five prefecture-level cities in Zhejiang Province (including Hangzhou, Ningbo, Shaoxing, Jiaxing, and Huzhou, as shown in Figure 1b), which is part of the Yangtze River Delta region. According to the data provided by the Zhejiang Statistics Bureau, the Hangzhou Bay area covers an area of 44,988 km^2^ in 2018, with a total resident population of 30.796 million. As part of the northern subtropical region, the Hangzhou Bay area is characterized by a mild and humid climate with abundant rainfall. The annual average temperature in the Hangzhou Bay area is between 15 and 20 °C. The landforms in the Hangzhou Bay area are diverse, dominated by hills and coastal plains, and the overall terrain is high in the west and low in the east. The land use of the Hangzhou Bay area in 2015 indicates that construction land was mainly concentrated in the coastal plain (Figure 1c).

As one of the demonstration areas of the reform and opening of China, its geographical conditions and policies make the Hangzhou Bay area an important growth pole for economic and social development not only in the Yangtze River Delta region but also in China as a whole [25]. However, in addition to rapid economic growth and a high urbanization rate, the Hangzhou Bay area has also experienced severe PM_2.5_ pollution in recent years [11]. In this study, the Hangzhou Bay area was selected as the study area to examine the spatiotemporal dynamics of the UPI effect within the context of rapid urban expansion.

### 2.2. Data Source

Several datasets, including PM_2.5_ data, land use data, administrative boundaries, historical Google Earth (Google, Mountain View, CA, USA) images, and digital elevation model (DEM, NASA, Washington, DC, USA) data, are listed in Table 1. The annual mean PM_2.5_ data were retrieved from the Atmospheric Composition Analysis Group at Dalhousie University (http://fizz.phys.dal.ca/~atmos/martin/). The dataset was obtained by combining the aerosol optical depth (AOD, NASA, Washington, DC, USA) retrievals from NASA Moderate-resolution Imaging Spectroradiometer (MODIS, NASA, Washington, DC, USA), Multi-angle Imaging Spectroradiometer (MISR, NASA, Washington, DC, USA), and Sea-viewing Wide Field-of-view Sensor (SeaWiFS, NASA, Washington, DC, USA) instruments with the GEOS-Chem (NASA, Washington, DC, USA) chemical transport model and subsequently calibrated against regional ground-based observations of both the total and compositional mass via geographically weighted regression (GWR, ESRI, Redlands, CA, USA). The ground-based PM_2.5_ measurements of mainland China were obtained from http://beijingair.sinaapp.com/, which were captured by individuals from instantaneous data records on the website of the Chinese Environmental Protection Agency [26,27].

The dataset has been documented with a suitable accuracy on a global scale (the cross-validated R^2^ value is 0.81) [28] and has been widely used [17,29]. Therefore, a subset covering the Hangzhou Bay area in 2000, 2005, 2010, and 2015 was adopted in this study. The data downloaded from the website have the World Geodetic System—the 1984 Coordinate System—with a spatial resolution of 0.01°. The projection transformation, extraction, and zonal statistics tools in geographical information systems (ESRI, Redlands, CA, USA) were implemented to successively process the annual mean PM_2.5_ concentration data.

Land use data and historical Google Earth images were used to extract the urban area, and historical Google Earth images were supplements to land use data. Unlike the other datasets, the source of historical Google Earth images is the Google Earth Pro software (Google, Mountain View, CA, USA). Moreover, administrative boundaries data and digital elevation model (DEM) data were used in this study.

## 3. Methods

### 3.1. Rationale

The rationale of this paper can be summarized as the emission, dispersion, and deposition of PM_2.5_. Principal component analysis performed on the aerosol data has revealed that vehicular emissions, coal/biomass combustion, industry sources, soil dust, and secondary formation are the main potential sources for the ionic components of PM_2.5_ in China [30]. The economic growth, urbanization, and industrialization of China have turned urban areas into the main sources and victims of air pollution [31].

By emitting the PM_2.5_ pollutant into the atmosphere, the process of dispersion results in transboundary air pollution. Dispersion is always accompanied by deposition, which is a process that removes pollutants from the atmosphere and transfers them to the ground surface. After emission, some of the particulates settle due to gravitation, some are eluted from the atmosphere by precipitation, and others remain in the atmosphere for a long time. It has been assumed that meteorological conditions, particle properties, and underlying surface characteristics can affect the dispersion and deposition of PM_2.5_ [32].

**Figure 2:**
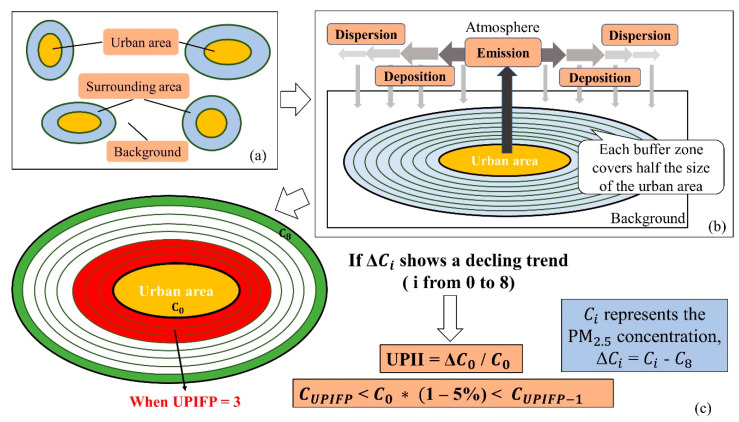
Model of the urban particulate matter island (UPI) effect. (**a**) Division of the study area; (**b**) the three processes of particulate emission, dispersion, and deposition; (**c**) evaluation criterion and two metrics of the UPI effect.

The UPI effect, referring to the phenomenon whereby the high PM_2.5_ concentrations in urban areas are gradually attenuated to surrounding areas, is essentially a comprehensive manifestation of the emission, dispersion and deposition of PM_2.5_ (Figure 2b).

### 3.2. Extraction of the Urban and Surrounding Areas

To determine the occurrence of the UPI effect, the study area was divided into urban, surrounding, and background areas (Figure 2a). Considering that the county/district of every prefecture-level city or municipality is the most basic economic and social unit in China, the urban area in this paper was defined as the urban land of every county or district in these five prefecture-level cities. Based on the land use data and historical Google Earth images, the urban areas in 2000, 2005, 2010, and 2015 were identified and extracted.

Specifically, for each urban area, eight buffer zones surrounding the urban area were generated using geographical information systems software, and each buffer zone covered half the size of the urban area. The purpose of the equal-area buffer zone instead of the equal-distance buffer zone is to ensure that each urban area is comparable. Based on both, the size of each urban area and the distance among the urban areas, eight buffer zones eventually proved to be the best choice. However, some urban areas were geographically very close, leading to overlapping buffer zones. Instead of removing these urban areas directly from this experiment as in a previous study [17], the method of merging was applied in this paper, which means that the urban areas with overlapping buffer zones were regarded as one urban area.

**Figure 3:**
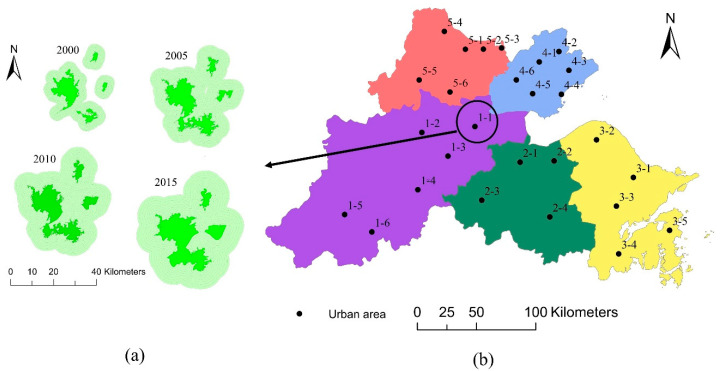
Urban areas in the Hangzhou Bay area. (**a**) Urban areas and buffer zones in Hangzhou (urban1-1) in 2000, 2005, 2010, and 2015; (**b**) location of the 27 urban areas.

To illustrate the above more clearly, the eight districts of Hangzhou (Shangcheng, Xiacheng, Jianggan, Gongshu, Xihu, Binjiang, Xiaoshan, and Yuhang) were selected as an example (Figure 3a). In 2000, Jianggan District and Yuhang District, located northeast of Hangzhou, were relatively far from the other six districts. However, with rapid urban expansion, overlapping buffer zones emerged in 2005. Therefore, all these districts were merged into one urban area. Based on this principle, there are 27 urban areas in the study area, and each urban area has its own corresponding number, such as 1-1 (the first number represents the prefecture-level city) (Figure 3b). In this paper, these numbers were adopted to represent the 27 urban areas in the following sections.

### 3.3. Model of the UPI Effect

The average PM_2.5_ concentration in each urban and buffer was calculated. For each urban area, ΔC_i_ was defined as the PM_2.5_ concentration difference between the i-th buffer zone C_i_ and the outermost buffer zone C_8_ (the 8th buffer zone). It was calculated with the following Equation (1):

ΔC_i_ = C_i_ − C_8_, i = {0, 1, 2…, 8} (1)

where C_0_ (when i = 0) is the PM_2.5_ concentration of the urban area, and C_i_ is the PM_2.5_ concentration of the i-th buffer zone. ΔC is a series of differential values for the urban area and all its buffer zones. The trends of ΔC for each urban area in 2000, 2005, 2010, and 2015 were analyzed to examine the attenuation of the PM_2.5_ concentration. The urban area whose PM_2.5_ concentration was gradually attenuated from the urban area to the surrounding buffer zones was regarded as an urban area experiencing the UPI effect.

To quantitatively delineate the UPI effect and its spatiotemporal pattern, two metrics have been defined. The first metric is the UPI intensity (UPII), which reflects the magnitude of the UPI effect and can be formulated in Equation (2) as follows:

UPII = ΔC_0_/C_0_ (2)

This means that the PM_2.5_ concentration ratio decayed from the urban area to the outermost buffer zone. In this paper, 0.1 is set as the UPII threshold. Considering PM_2.5_ standard from the World Health Organization (WHO), urban areas with C_0_ > 10 μg/m3 and UPII > 0.1 are regarded as those with a high UPII. It is well known that urban pollution stems from both natural and anthropogenic sources [33]. Therefore, by removing the influence of the natural background, UPII can be viewed as the urban pollution caused by anthropogenic activities [17].

The second metric, the UPI footprint (UPIFP), was defined as the number of buffer zones across which the urban PM_2.5_ concentration decreases by 5% (half of the UPII threshold) from the urban area. It can be calculated in Equation (3) as follows:

C_UPIFP_ < C_0_ × (1 − 5%) < C_UPIFP-1_ (3)

UPIFP quantifies the extent of the continuous effects from the urban area to the surrounding areas. In this paper, UPIFP is an integer ranging from 1 to 8 (Figure 2c). A higher UPIFP value indicates that urban pollution is transported to a further buffer zone. In other words, the higher the value of UPIFP is, the lower the degree of PM_2.5_ accumulation in the urban area is compared to that in the surrounding area, and the less difficult it is for PM_2.5_ pollution to spread outward. In this paper, urban areas with UPIFP values higher than 4 are regarded as areas with a large pollution PM_2.5_ effect range.

The overall concept of this paper is shown in Figure 2. Based on the rationale mentioned above, the method can be defined as according to several steps: (1) extract the urban areas; (2) generate the equal-area buffer zones as the surrounding areas; (3) calculate the average PM_2.5_ concentration in each urban and surrounding area; (4) analyze the concentration difference from the urban area to the outermost buffer zone; (5) determine whether the UPI phenomenon exists or not; and (6) if so, calculate the UPII and UPIFP of each urban area.

## 4. Results

### 4.1. Spatial Patterns of Urban Expansion

**Figure 4:**
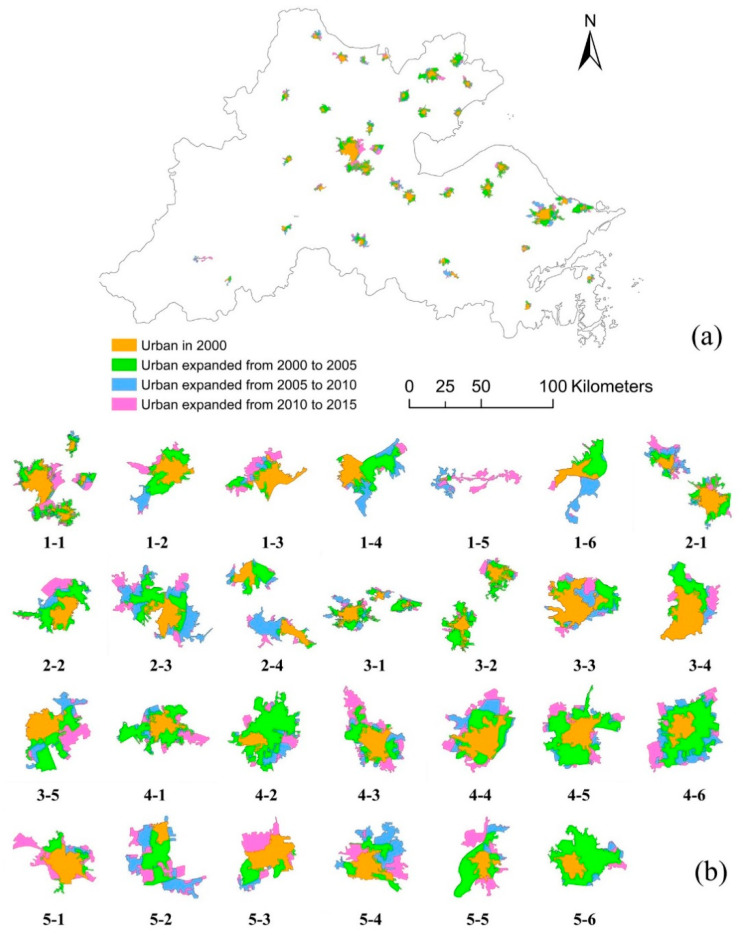
Urban expansion from 2000 to 2015 in the Hangzhou Bay area. (**a**) All urban areas; (**b**) each urban area.

**Figure 5:**
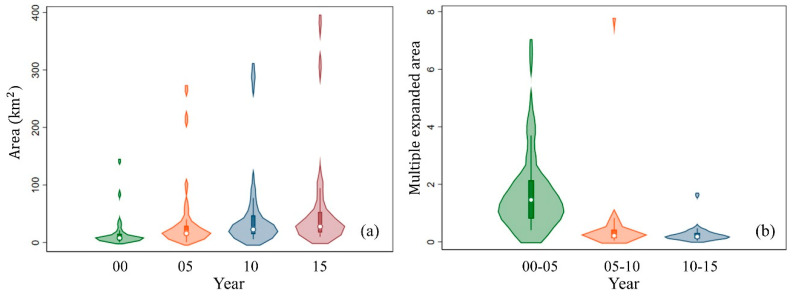
Statistics on the urban expansion of the 27 urban areas in the Hangzhou Bay area. (**a**) Area in 2000, 2005, 2010, and 2015; (**b**) multiple expanded areas during 2000–2005, 2005–2010, and 2010–2015.

The urban expansion that occurred in the Hangzhou Bay area from 2000 to 2015 is shown in Figure 4a. The total area of the extracted urban areas was 470.41 km^2^ in 2000, 1087.96 km^2^ in 2005, 1325.15 km^2^ in 2010, and 1584.68 km^2^ in 2015. The expansion that occurred from 2000 to 2005 occupied 55.42% of the total urban expansion from 2000 to 2015, making 2000–2005 the period with the most rapid urban growth in the Hangzhou Bay area, followed by 2010–2015 (23.29%), and the period with the least rapid growth was 2005–2010 (21.29%). Violin plots of the study area and multiple expanded areas are shown in Figure 5a,b, respectively. Combining the kernel density estimation curve and box plot, the violin plots indicated that the median area increased from 2000 to 2015, while the median expanded area decreased over this period. Although violin plots are less common than box plots, they show similar information. In addition to the median and interquartile range, a violin plot shows the distribution of the data with a rotated smoothing curve.

To visualize the expansion of each urban area more clearly, the specific expansion of each urban area is shown in Figure 4b (the scale of each urban area varies). Each urban area expanded from 2000 to 2015, but considering the expansion form and size, each urban area differed. In terms of the expansion form, basically, every urban area expanded outward from the original urban area (referring to the urban area in 2000) in all directions over this period without terrain and hydrological restrictions. In addition, the area of each urban area was analyzed as well. According to the calculation of the area, urban1-1 (which is the urban area whose corresponding number is 1-1, the same as below) possessed the largest area all the time, followed by urban3-1. Four urban areas expanded more than three times from 2000 to 2005, i.e., urban4-6 expanded by 348.31%, urban4-2 expanded by 659.02%, urban5-2 expanded by 369.92%, and urban5-6 expanded by 449.12%.

### 4.2. PM_2.5_ Concentration in the Urban Areas and Buffer Zones

**Figure 6:**
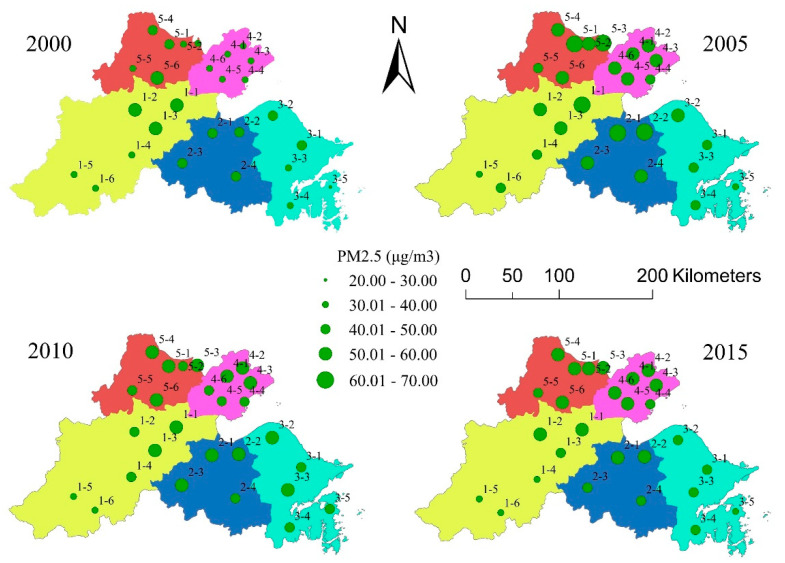
The annual mean fine particulate matter (PM_2.5_) concentration in each urban area.

The annual mean PM_2.5_ concentration within each urban area in 2000, 2005, 2010, and 2015 is shown in Figure 6. From the perspective of the spatial distribution, both the northeast and southwest regions of the Hangzhou Bay area presented relatively low PM_2.5_ concentrations. In terms of the PM_2.5_ concentration of each urban area, the PM_2.5_ concentration in urban1-4, urban1-5, urban1-6, urban3-4, urban3-5, and urban5-5 remained low from 2000 to 2015, while that in urban1-1 remained high throughout in this period. In addition, the cluster formed by urban4-1, urban4-2, urban4-3, urban4-4, urban4-5, and urban4-6 experienced a notable increase in PM_2.5_ concentration from 2000 to 2015. These six urban areas are all located in Jiaxing and are geographically close to one another.

By evaluating the PM_2.5_ concentrations in these four years, the most significant point is that the PM_2.5_ concentrations in 2000 were considerably lower than that in the other three years. The minimum concentration in 2000 was 25.81 μg/m^3^, while it was 36.12 μg/m^3^ in 2005, 36.37 μg/m^3^ in 2010 and 34.16 μg/m^3^ in 2015. At the same time, the maximum concentration in 2000 was 54.03 μg/m^3^, while it was 65.75 μg/m^3^ in 2005, 59.31 μg/m^3^ in 2010, and 58.57 μg/m^3^ in 2015. In summary, both the minimum and maximum concentrations were the lowest in 2000.

**Figure 7:**
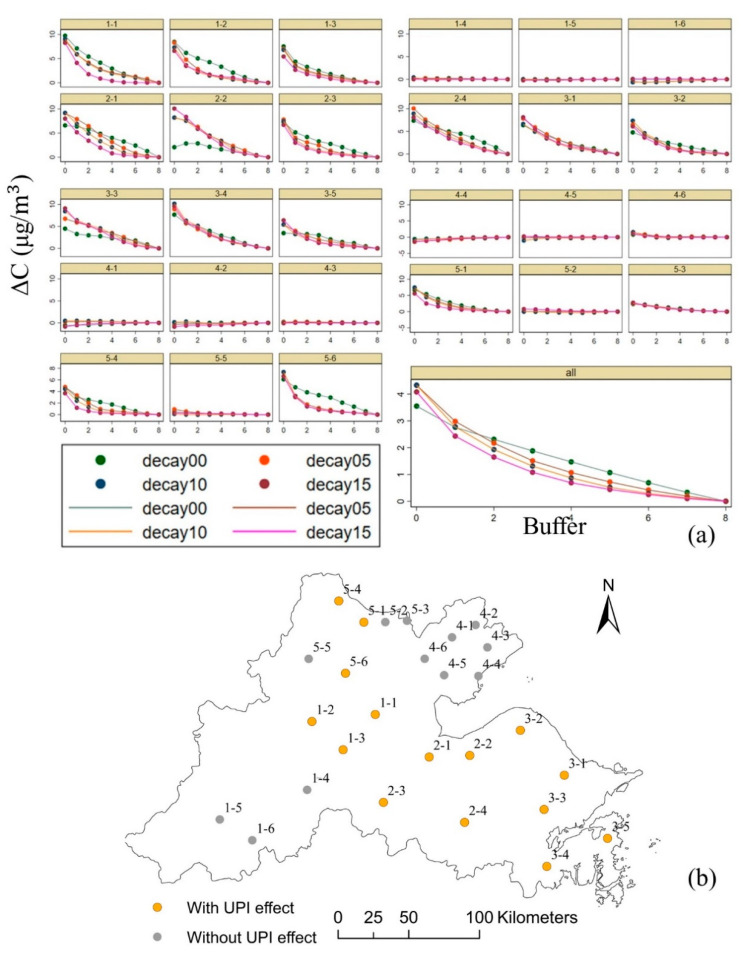
The occurrence of the UPI effect. (**a**) Trends of concentration difference (ΔC) from the urban area to the buffer zones for the 27 urban areas in the Hangzhou Bay area in 2000, 2005, 2010, and 2015; (**b**) spatial distribution of the urban areas with the UPI effect.

Regarding each urban area, ΔC (the PM_2.5_ concentration difference between the urban area/buffer zone and the outermost buffer zone) of the 27 urban areas in 2000, 2005, 2010, and 2015 are shown in Figure 7a. In 15 of the 27 urban areas, ΔC clearly decayed from the urban area to the buffer zones, including urban1-1, urban1-2, urban1-3, urban2-1, urban2-2, urban2-3, urban2-4, urban3-1, urban3-2, urban3-3, urban3-4, urban3-5, urban5-1, urban5-4, and urban5-6, which were regarded as urban areas with UPI effects in this paper. The distribution of the urban areas with the UPI effect is shown in Figure 7b. Except for urban2-2, for each of the remaining 14 urban areas, the shapes of the four curves are roughly similar.

When ΔC of all 27 urban areas are combined (labeled “all” in Figure 7a), the four decay curves for 2000, 2005, 2010, and 2015 can be obtained. Except for 2000, the curves for the other three years are very similar in both shape and value, and their ΔC_0_ values are all higher than 4. The slope of the attenuation decreases with the distance in 2005, 2010, and 2015. Conversely, ΔC_0_ in 2000 is 3.55, which is slightly lower than that in the other three years, and the slope of the attenuation remains essentially the same.

### 4.3. Spatiotemporal Dynamics of UPII and UPIFP

**Figure 8:**
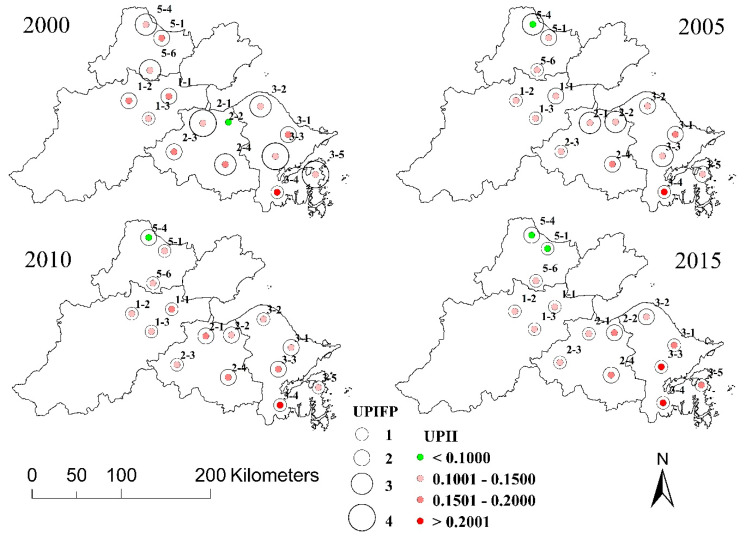
Spatial distribution of the UPI intensity (UPII) and UPI footprint (UPIFP) for the urban areas with UPI effects in 2000, 2005, 2010, and 2015.

**Figure 9:**
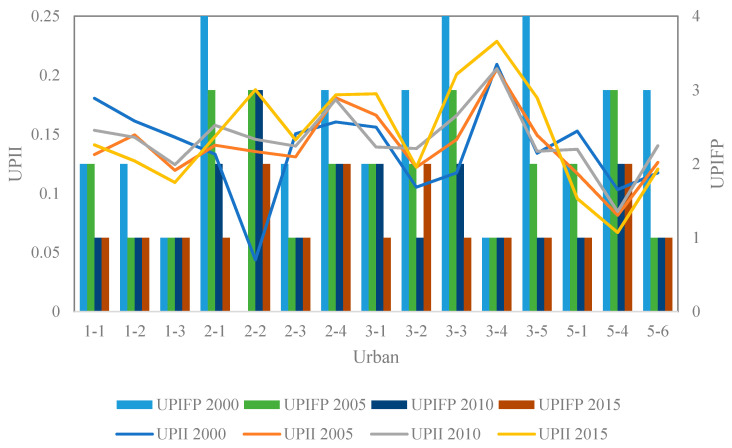
UPI intensity (UPII) and UPI footprint (UPIFP) in 2000, 2005, 2010, and 2015.

The spatiotemporal dynamics of the UPII and UPIFP for the urban areas with UPI effects in the Hangzhou Bay area are shown in Figure 8. For comparison purposes, the UPII in the four years are all classified with 0.1, 0.15, and 0.2. Overall, the UPII in each urban area remained relatively stable over time (Figure 9). From the perspective of the spatial distribution, the urban areas with a higher UPII were distributed in the central and southeast regions of the Hangzhou Bay area. The urban area with the highest UPII was urban3-4, whose UPII exceeded 0.2 every year. In addition, the UPII of urban1-1, urban1-2, urban1-3, urban2-1, urban2-3, urban2-4, urban3-1, urban3-2, urban3-3, urban3-5, and urban5-6 were higher than 0.1 in these four years.

In contrast to the UPII, not all urban areas have a UPIFP. Among the 15 urban areas, 14 urban areas have a UPIFP all four years, while urban2-2 only has a UPIFP in 2005, 2010, and 2015. The UPIFP in 2000 includes 1, 2, 3, and 4. In 2005 and 2010, 1, 2, and 3 still occurred, while in 2015, only 1 and 2 remained. The most notable UPIFP phenomenon is that from 2000 to 2015, the UPIFP generally showed a downward trend, especially from 2000 to 2005, which implied that compared to the surrounding area, the PM_2.5_ accumulation in the urban area became more severe in this period.

## 5. Discussion

### 5.1. Emissions

**Figure 10:**
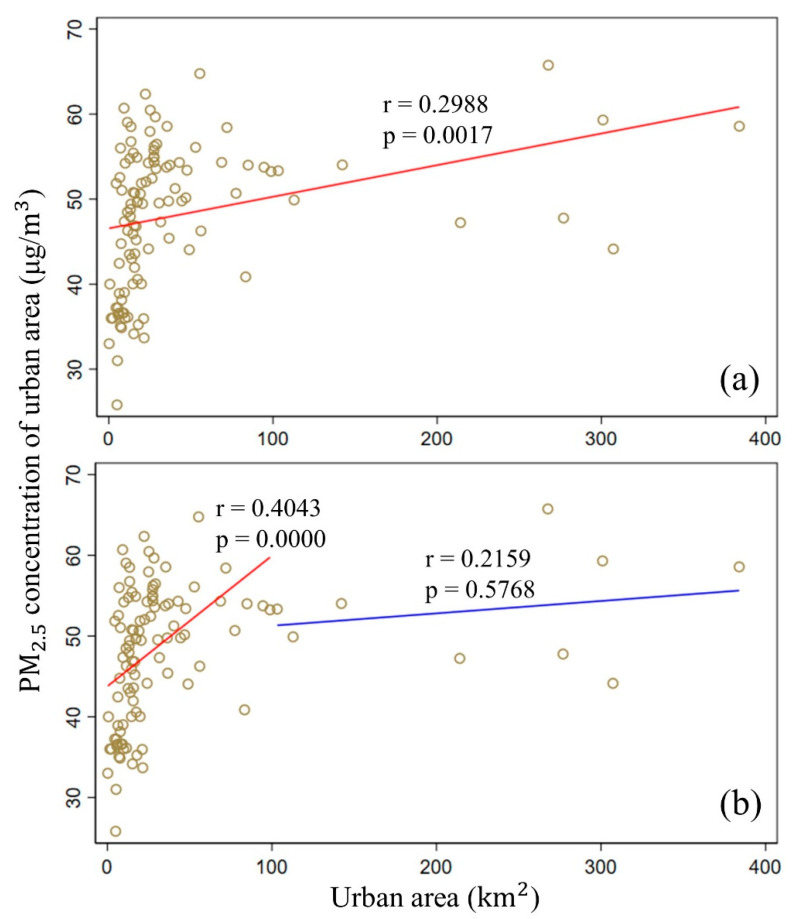
Relationship between the area and PM_2.5_ concentration in the urban areas. (**a**) All urban areas; (**b**) urban areas divided at an area of 100 km^2^.

In this paper, urban areas are considered the source and container of PM_2.5_ pollution, and the PM_2.5_ concentration in the urban areas is directly related to local emissions. Because it is the result of economic growth, urban expansion can reflect the development of cities to a certain extent. Pearson correlation analysis between the area and PM_2.5_ concentration of the urban areas indicates that there exists a positive correlation between the area and concentration, especially when the area is smaller than 100 km^2^ (Figure 10). This is consistent with previous observations [29]. However, the environmental Kuznets curve (EKC) hypothesis claims that there exists an inverted U-shaped connection between environmental degradation and economic growth [34], which means that the Hangzhou Bay area is still at the initial stage of economic growth. Therefore, although PM_2.5_ pollution can be fundamentally reduced by controlling emissions in the short term, it comes at a significant cost and requires efforts from both the local government and polluting companies [35]. In addition, studies have shown that urban form can influence air pollution in China, and moderately scattered and polycentric urban development is encouraged [36].

### 5.2. Dispersion and Deposition

**Figure 11:**
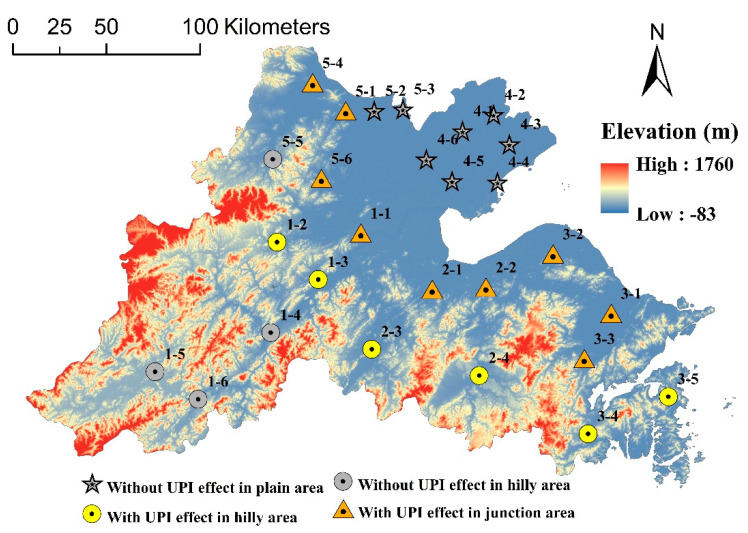
Division of the urban areas by the terrain.

With increasing economic development and urbanization, the underlying surface of dispersion and deposition has changed. On the one hand, due to the rapid urban expansion, parts of the former surrounding area have become urban areas, and parts of the background area have been transformed into surrounding areas. On the other hand, the land use of the surrounding and background areas has also changed in this period, such as the transition from farmland to rural settlements. To further examine the mechanism behind the UPI effect, these 27 urban areas were divided into three categories (Figure 11).

Plain area: the urban areas were distributed in clusters with a high integrity. Due to the flat terrain, cities were generally densely distributed, and urban expansion resulted in a major reduction in the distance between neighboring urban areas, which might enhance the interaction of pollution. In addition, the surrounding and background areas were usually dominated by cultivated land and rural settlements. Therefore, the PM_2.5_ concentration in the plain area was relatively even, and the probability of the UPI effect was relatively low in this area.

Hilly area: the urban areas were scattered with notable independence. Because of the woodlands in the surrounding areas, the pollutants emitted from the urban areas could be effectively settled. In addition, the large forests in the background areas diminished the effect of urban expansion on the distance between the urban areas. However, due to the terrain, urban expansion was limited, which might make urban pollution accumulation less distinct. Therefore, the probability of the UPI effect was relatively random in this area, and the decrease in UPIFP might be due to the shorter distance between the urban boundaries and woodlands.

Junction of plains and hills: the urban areas were zonally distributed, with both integrity and independence. Compared with the urban areas surrounded by hills, the expansion was relatively less restricted by the terrain but more susceptible to the influence of neighboring urban areas. Compared with the plain area, the surrounding woodlands could effectively settle pollutants. In this paper, the urban areas located in the junction area exhibited a high probability of the UPI effect, and the decrease in UPIFP was also due to the shorter distance between the urban boundaries and woodlands.

### 5.3. Main Findings

In this paper, we proposed a framework to characterize the UPI effect of the Hangzhou Bay area during 2000–2015. The urban areas experienced a rapid expansion during this period. However, the existence of the UPI effect in each urban area differed slightly. More than half of the urban areas exhibited the UPI effect, and the existence of the UPI effect was not related to the PM_2.5_ concentration, but instead to the terrain, and urban areas could be classified into three categories: plain areas, hilly areas, and the junction of plains and hills. Terrain not only affects the dispersion and deposition of pollutants, but also affects the expansion of urban areas, which is the source of emissions. For those urban areas with the UPI effect, the UPII value in each urban area remained relatively stable over time. The UPIFP value generally showed a downward trend, implying that compared to the surrounding areas, PM_2.5_ accumulation in the urban areas became more severe in this period. Compared with existing literature, at the national level, the concentration of monitoring sites around and within the cities were highly correlated [37]. Remote sensing data have also been used to measure the difference in the PM_2.5_ concentration between urban and non-urban areas, but without the concentration trends [38]. Some studies focused on only a single megacity: there was no significant difference in concentration between the urban and suburban areas of Shanghai [39]; however, the difference was obvious in Beijing [40]. In this paper, the study area is an urban agglomeration, which between a nation and a single city. Previous studies have generated equidistant buffer zones around cities but did not measure the specific concentration trends of PM_2.5_ [11]. The impact of land use on the spatial distribution of concentration was previously studied, and forests were found to have the greatest impact on PM_2.5_ removal [11,41,42], which is consistent with the results in this paper. In general, the results of this paper are consistent with existing literature, and the urban-rural concentration trends on the district/county scale in this paper can provide a theoretical basis for pollution control during urban agglomeration.

## 6. Conclusions

The UPI effect, which refers to the phenomenon whereby the high particle concentration in the urban areas is gradually attenuated to the surrounding areas, was defined and investigated in this paper. The existing literature has revealed that the rationale for the UPI effect is insufficient, and the mutual influence between cities was often ignored. Therefore, this study tried to fill the gap by dividing the study area into urban, surrounding, and background areas and by dividing the dynamic processes of pollutants into emission, dispersion, and deposition. Moreover, to better study the UPI effect at the local level, the urban area in this paper was changed from the urban land of every prefecture to every county or district. As an area with many cultural heritages, the Hangzhou Bay area was selected as the study area. Based on the observations in the 27 urban areas in the Hangzhou Bay area from 2000 to 2015, we examined the urban expansion and the occurrence of the UPI effect for each urban area. The main findings are as follows:

1. Every urban area in the Hangzhou Bay area experienced rapid expansion from 2000 to 2015. For most of the urban areas, 2000–2005 was the period with the most rapid expansion, and several urban areas even attained growth rates above 300%. Although the form and magnitude of urban expansion varied, single-core expansion occurred in most urban areas.
2. By comparison, the PM_2.5_ concentrations in the urban areas in 2000 were considerably lower than that in 2005, 2010, and 2015. In addition, from the perspective of the spatial distribution, spatial heterogeneity of the PM_2.5_ concentrations were observed in the Hangzhou Bay area. Overall, the PM_2.5_ concentration in the urban area had a positive correlation with its area.
3. More than half of the urban areas exhibited the UPI effect in the Hangzhou Bay area during this period. Most urban areas with the UPI effect had a relatively high and stable UPII as well. In contrast to the UPII, the UPIFP values showed a downward trend along with urban expansion.
4. The urban areas could be divided into three categories according to the terrain, i.e., plain areas, hilly areas, and the junction of plains and hills, and the probability of the UPI effect for the different categories varied notably.

Overall, this paper can compensate for the lack of research on the UPI effect at the local level and provide scientific evidence for air pollution control during urban agglomeration planning.

## Figures and Tables

**Table 1:** Description of the data used in this study.

Data	Data Description	Source
PM_2.5_ data	Annual mean PM_2.5_ concentration in China with a spatial resolution of 0.01° × 0.01° in 2000–2015	http://fizz.phys.dal.ca/~atmos/martin/
Land use data	Land use data in 2000, 2005, 2010, and 2015 with a spatial resolution of 30 m × 30 m	http://nnu.geodata.cn:8008/
Administrative boundaries	Shape files of prefectures and counties in Zhejiang Province	http://www.resdc.cn/
Historical Google Earth images	Google Earth images of the Hangzhou Bay area in 2000, 2005, 2010, and 2015	Download the software from https://www.google.com/earth/versions/#earth-pro and install it, then follow these instructions: https://support.google.com/earth/answer/148094?hl=en/
Digital elevation model (DEM) data	ASTER (Advanced Spaceborne Thermal Emission and Reflection Radiometer) GDEM (Global Digital Elevation Model) data with a spatial resolution of 30 m × 30 m	https://lpdaac.usgs.gov/
